# Supplementary material for: The Function of Flavonoids in the Diurnal Rhythm under Rapidly Changing UV Conditions—A Model Study on Okra
Source: Plants (Basel). 2021 Oct 22;10(11):2268. doi: 10.3390/plants10112268 (PMC8624821; doi:10.3390/plants10112268)
Supplement: Supplementary file 1 [file plants-10-02268-s001.zip › plants-1404455-supplementary.pdf]

Experiment 1

+UV

-UV

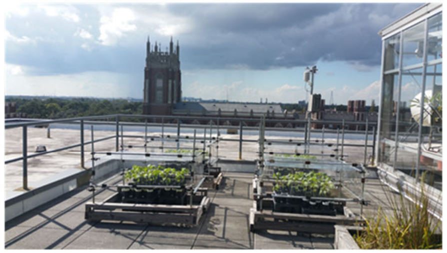

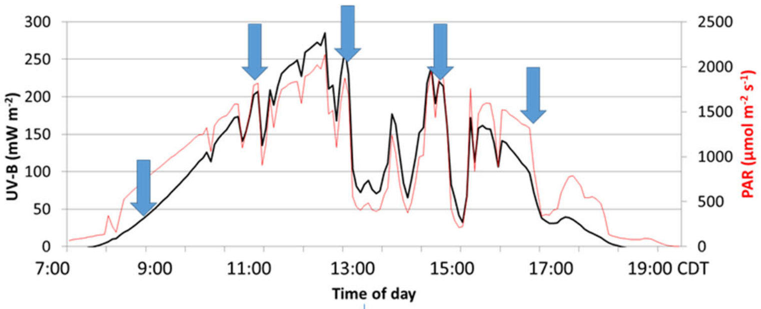

Plant morphology

Flavonoid content optically
UV-shielding from extracts
Antioxidant activity from extracts
Soluble phenolics from extracts

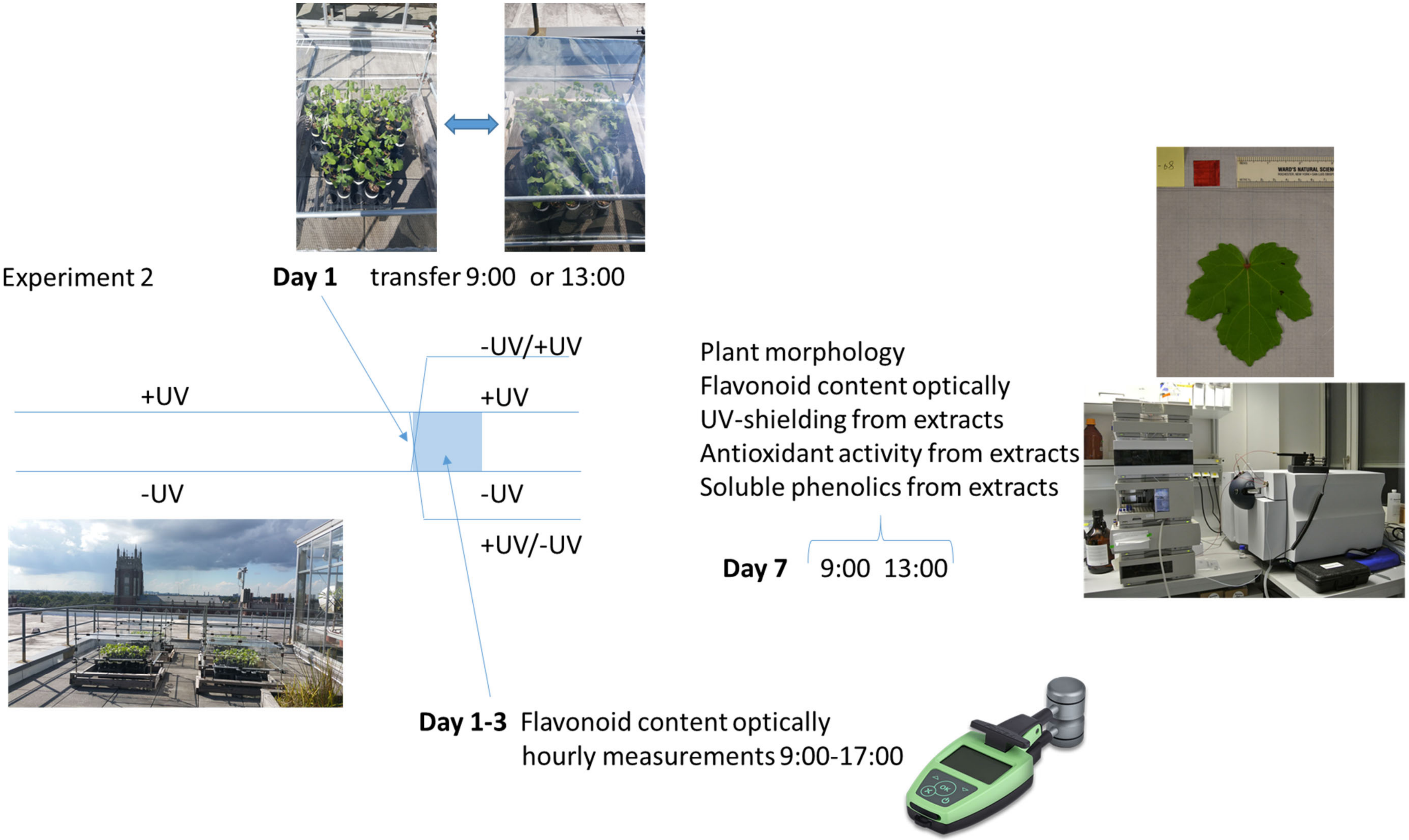

Fig S1: Experimental set up for experiment 1 (for studying the diurnal rhythm) and experiment 2 (for studying changes in the diurnal rhythm due to the transfer to the opposite UV condition).

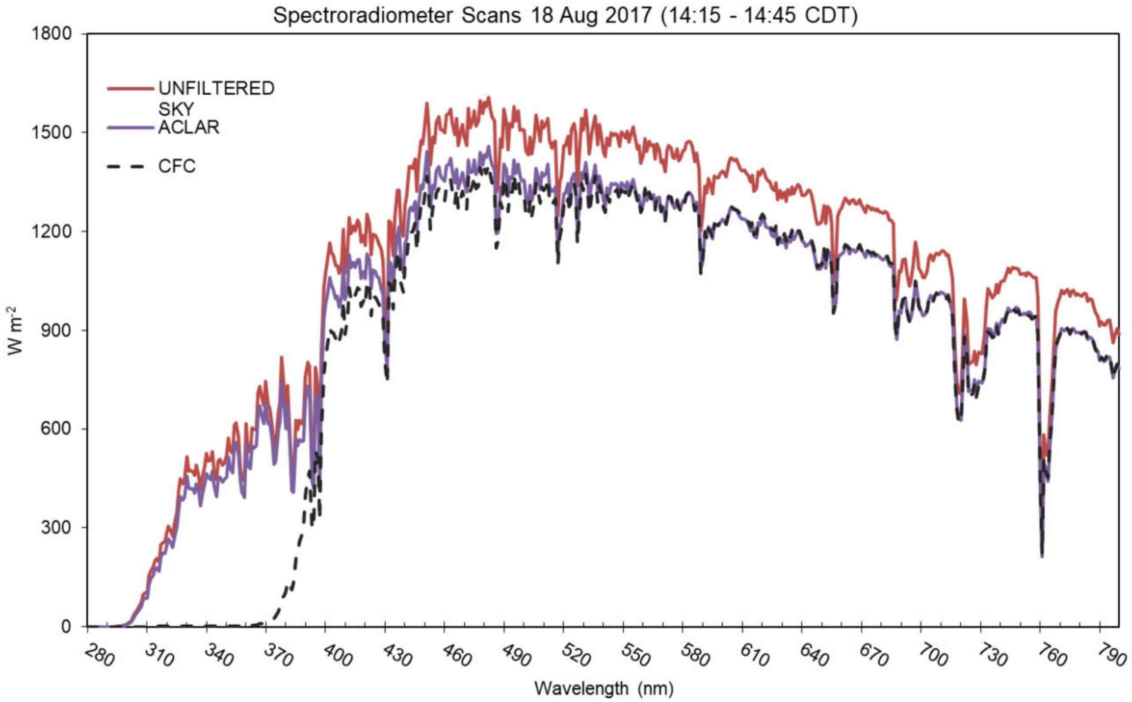

Figure S2. Representative clear-sky spectral irradiance for the +UV treatment (ACLAR film), -UV treatment (CFC) film and unfiltered solar radiation.

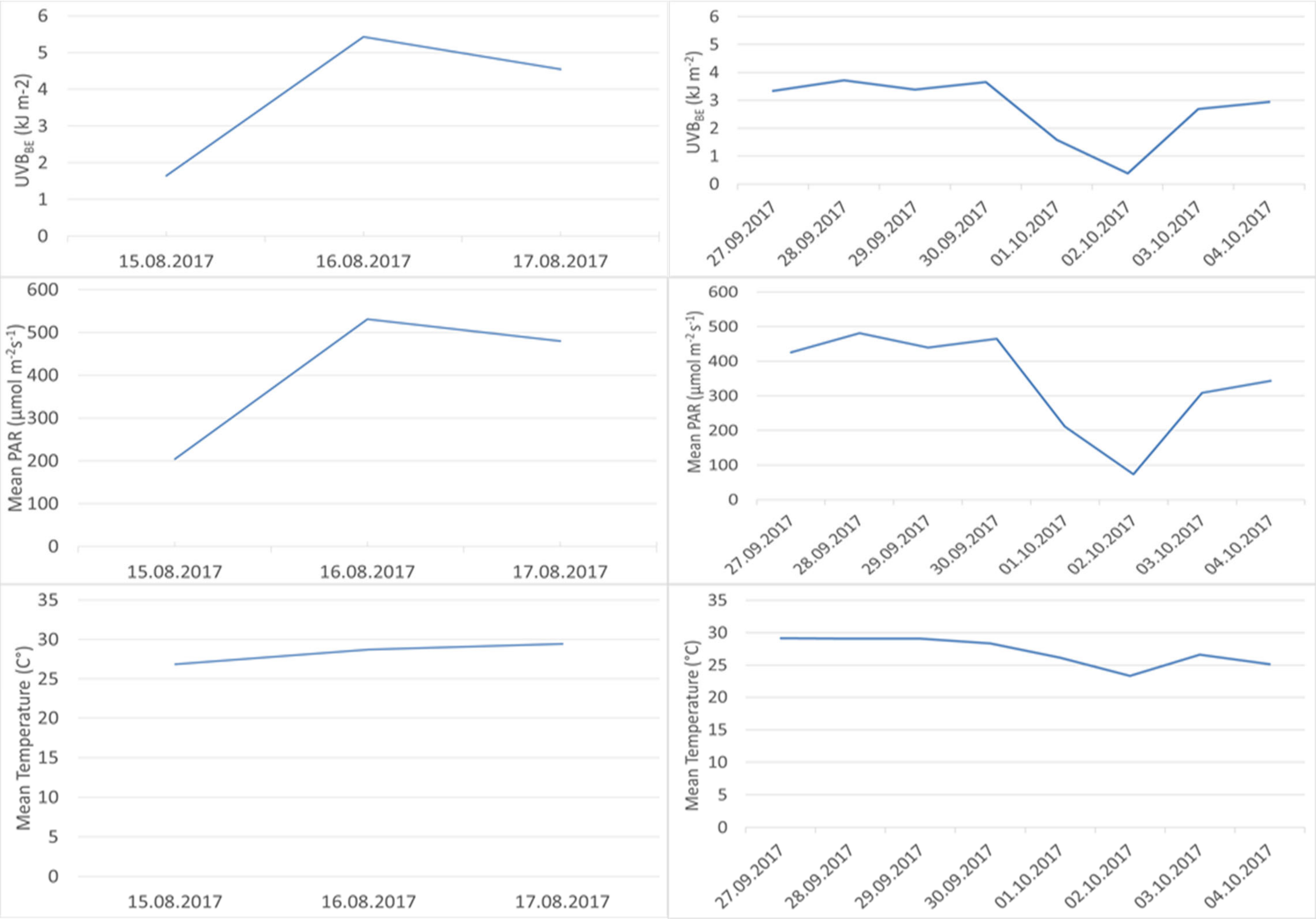

Fig S3: Environmental conditions during experiment 1 (left) and experiment 2 (right)
